# Supplementary material for: Altered thymic niche synergistically drives the massive proliferation of malignant thymocytes
Source: eLife. 2025 Sep 16;13:RP101137. doi: 10.7554/eLife.101137 (PMC12440356; doi:10.7554/eLife.101137)
Supplement: Figure 5—source data 1. — Values reported in figures are the adjusted p-values. Significance threshold was set to 0.05. Data rounded to 4 decimal places. [file elife-101137-fig5-data1.docx]

#### Figure 5 - Source data 1: Detailed statistics for phenotype comparison using Fisher’s exact test and pairwise comparisons; adjusted p values were calculated with the Benjamini & Hochberg method. Values reported in figures are the adjusted p values. Significance threshold was set to 0.05. Data rounded to 4 decimal places.

| **Group 1** | **Group 2** | **p.value** | **significant** | **p.value adjusted** | **significant adjusted** |
| --- | --- | --- | --- | --- | --- |
| WT | lck:NICD | 1 | FALSE | 1 | FALSE |
| WT | ccl25a:il7 | 0.0486 | TRUE | 0.0694 | FALSE |
| WT | lck:NICD-MycN | 0.0007 | TRUE | 0.0017 | TRUE |
| WT | ccr9a:il7r | 0.0002 | TRUE | 0.0006 | TRUE |
| WT | ccr9a:il7r + ccl25a:il7 | 4.90E-06 | TRUE | 2.20E-05 | TRUE |
| WT | lck:NICD-MycN + ccl25a:il7 | 4.15E-08 | TRUE | 4.67E-07 | TRUE |
| WT | ccr9a:il7r^NPC^ | 8.70E-09 | TRUE | 1.31E-07 | TRUE |
| WT | lck:NICD-MycN + ccr9a:il7r | 2.70E-07 | TRUE | 2.03E-06 | TRUE |
| WT | lck:NICD-MycN + ccr9a:il7r^NPC^ | 3.00E-12 | TRUE | 1.47E-10 | TRUE |
| lck:NICD | ccl25a:il7 | 0.0730 | FALSE | 0.0996 | FALSE |
| lck:NICD | lck:NICD-MycN | 0.0040 | TRUE | 0.0076 | TRUE |
| lck:NICD | ccr9a:il7r | 0.0011 | TRUE | 0.0024 | TRUE |
| lck:NICD | ccr9a:il7r + ccl25a:il7 | 5.2885E-05 | TRUE | 0.0002 | TRUE |
| lck:NICD | lck:NICD-MycN + ccl25a:il7 | 6.92E-07 | TRUE | 4.42E-06 | TRUE |
| lck:NICD | ccr9a:il7r^NPC^ | 9.67E-08 | TRUE | 8.70E-07 | TRUE |
| lck:NICD | lck:NICD-MycN + ccr9a:il7r | 1.59E-06 | TRUE | 7.93E-06 | TRUE |
| lck:NICD | lck:NICD-MycN + ccr9a:il7r^NPC^ | 1.24E-10 | TRUE | 2.78E-09 | TRUE |
| ccl25a:il7 | lck:NICD-MycN | 0.4027 | FALSE | 0.4531 | FALSE |
| ccl25a:il7 | ccr9a:il7r | 0.2344 | FALSE | 0.2776 | FALSE |
| ccl25a:il7 | ccr9a:il7r + ccl25a:il7 | 0.0351 | TRUE | 0.0526 | FALSE |
| ccl25a:il7 | lck:NICD-MycN + ccl25a:il7 | 0.0025 | TRUE | 0.0049 | TRUE |
| ccl25a:il7 | ccr9a:il7r^NPC^ | 0.0008 | TRUE | 0.0018 | TRUE |
| ccl25a:il7 | lck:NICD-MycN + ccr9a:il7r | 0.0007 | TRUE | 0.0017 | TRUE |
| ccl25a:il7 | lck:NICD-MycN + ccr9a:il7r^NPC^ | 8.65E-06 | TRUE | 3.54E-05 | TRUE |
| lck:NICD-MycN | ccr9a:il7r | 0.0872 | FALSE | 0.1155 | FALSE |
| lck:NICD-MycN | ccr9a:il7r + ccl25a:il7 | 0.1314 | FALSE | 0.1642 | FALSE |
| lck:NICD-MycN | lck:NICD-MycN + ccl25a:il7 | 0.0493 | TRUE | 0.0694 | FALSE |
| lck:NICD-MycN | ccr9a:il7r^NPC^ | 0.0170 | TRUE | 0.0283 | TRUE |
| lck:NICD-MycN | lck:NICD-MycN + ccr9a:il7r | 0.0281 | TRUE | 0.0436 | TRUE |
| lck:NICD-MycN | lck:NICD-MycN + ccr9a:il7r^NPC^ | 0.0003 | TRUE | 0.0007 | TRUE |
| ccr9a:il7r | ccr9a:il7r + ccl25a:il7 | 0.2187 | FALSE | 0.2660 | FALSE |
| ccr9a:il7r | lck:NICD-MycN + ccl25a:il7 | 0.0003 | TRUE | 0.0007 | TRUE |
| ccr9a:il7r | ccr9a:il7r^NPC^ | 6.7619E-05 | TRUE | 0.0002 | TRUE |
| ccr9a:il7r | lck:NICD-MycN + ccr9a:il7r | 8.0909E-05 | TRUE | 0.0003 | TRUE |
| ccr9a:il7r | lck:NICD-MycN + ccr9a:il7r^NPC^ | 7.85E-07 | TRUE | 4.42E-06 | TRUE |
| ccr9a:il7r + ccl25a:il7 | lck:NICD-MycN + ccl25a:il7 | 0.0258 | TRUE | 0.0415 | TRUE |
| ccr9a:il7r + ccl25a:il7 | ccr9a:il7r^NPC^ | 0.0135 | TRUE | 0.0234 | TRUE |
| ccr9a:il7r + ccl25a:il7 | lck:NICD-MycN + ccr9a:il7r | 0.0078 | TRUE | 0.0141 | TRUE |
| ccr9a:il7r + ccl25a:il7 | lck:NICD-MycN + ccr9a:il7r^NPC^ | 0.0016 | TRUE | 0.0033 | TRUE |
| lck:NICD-MycN + ccl25a:il7 | ccr9a:il7rNPC | 0.7902 | FALSE | 0.8269 | FALSE |
| lck:NICD-MycN + ccl25a:il7 | lck:NICD-MycN + ccr9a:il7r | 0.6492 | FALSE | 0.6955 | FALSE |
| lck:NICD-MycN + ccl25a:il7 | lck:NICD-MycN + ccr9a:il7r^NPC^ | 0.1243 | FALSE | 0.1598 | FALSE |
| ccr9a:il7r^NPC^ | lck:NICD-MycN + ccr9a:il7r | 0.9308 | FALSE | 0.9519 | FALSE |
| ccr9a:il7r^NPC^ | lck:NICD-MycN + ccr9a:il7r^NPC^ | 0.4821 | FALSE | 0.5291 | FALSE |
| lck:NICD-MycN + ccr9a:il7r | lck:NICD-MycN + ccr9a:il7r^NPC^ | 0.3705 | FALSE | 0.4275 | FALSE |

#### 
